# Harm Reduction and Decriminalization of Sex Work: Introduction to the Special Section

**DOI:** 10.1007/s13178-021-00636-0

**Published:** 2021-10-16

**Authors:** Belinda Brooks-Gordon, Max Morris, Teela Sanders

**Affiliations:** 1 School of Psychological Sciences, University of London, BirkbeckLondon, UK; 2 Department of Criminology, Kingston University, Kingston, UK; 3 Department of Criminology, Leicester University, Leicester, UK

**Keywords:** Sex work, Harm reduction, Decriminalization, Sex worker, Safety

## Abstract

**Introduction:**

This special section of *Sexuality Research and Social Policy*, edited by Belinda Brooks-Gordon, Max Morris and Teela Sanders, has its origins in a colloquium sponsored by the University of Cambridge Socio-Legal Group in 2020. The goal was to promote the exchange of ideas between a variety of disciplinary research fields and applied perspectives on harm reduction and the decriminalization of sex work. The colloquium took place during the emergence of the coronavirus pandemic in February 2020.

**Methods:**

We explore the impact of Covid-19 on understandings of sex work, outline the basic underpinning legal philosophical question, explore the intersectional politics of decriminalization, summarize contemporary international health and human rights campaigns, explore contemporary public opinion trends on the issue, and illustrate the universal principles. Finally, we summarize the special section papers (N=12).

**Results:**

The Covid pandemic provided a lens through which to analyse the changes that have occurred in sex work and sex work research in the past decade and it also exacerbated intersecting inequalities, accelerated many social shifts already in motion whilst changing the course of others. In combination the papers in this special issue examine sex work policy and research across 12 countries in four continents to provide and important space for international and cross-cultural comparison.

**Conclusions:**

We present the timely contributions of diverse authors and comment on the significance of their research projects which support a decriminalization policy agenda for the benefit of academics, policymakers and practitioners to improve public health strategies and international responses.

**Policy Implications:**

The research here amplifies the focus on harm reduction and strengthens the case for public policy that decriminalizes commercial sex between consenting adults as the best strategy to reduce harm.

## The Impact of the COVID-19 Pandemic on Understandings of Sex Work

A major shift that the pandemic accelerated was the move to online working patterns, a trend that had been occurring in sex work markets over the past two decades (Bernstein, 2007; Jones, 2015, 2020; Sanders et al., 2018). This shift included those performing non-contact sex work and those moving into online sex work for the first time, leading to an increase in the marketing of online sex work. Given the economic shock of the pandemic, it was also unsurprising that both online and in-person sex work continued to take place during national lockdowns, despite emergency laws which reportedly sought to restrict social and sexual contact. In the UK context, the economic impact could be seen in the number of people claiming welfare for the first time and the reported difficulty of claiming state benefits (Bambra et al., 2020). These are circumstances known to lead to an increase in the proportion of people in sex work, especially when poverty becomes feminized (e.g. Glendinning & Miller, 1992). Jobs in service and hospitality sectors were among the most badly hit, at least initially. Some jobs will never return as furlough schemes end, businesses close and more people turn to various types of sex work, whether in-person or online, a framework to ensure that people can work in safety is required.

In addition to lockdowns, curfews, mobility restrictions, physical distancing, mask-wearing, quarantine and self-isolation, other measures were enforced or encouraged in response to COVID-19 and, in part, continue to be our ordinary lives. Framed in terms of necessary public health interventions during the pandemic, lockdown policies adopted in many nation states provided a natural experiment that separated the variable of ‘working in isolation’ and its resulting impacts such as loneliness and mental health deterioration from other variables, such as ‘type of work’, ‘income’ or ‘class’ and enabled a re-analysis of work that has conflated harms and poorer mental health outcomes with sex work *per se*.

For example, a systematic review of general social isolation and loneliness by Leigh-Hunt et al. (2017) found associations with poorer mental and physical health was corroborated by cross-sectional research from the COVID-19 psychological well-being study during the first year of the pandemic (Groake et al., 2020). These studies show that risk factors for loneliness were younger age group, separated or divorced, greater emotion regulation difficulties and poor quality of sleep due to the crisis, whereas protective factors were higher levels of social support, being married/cohabiting and the companionship of a greater number of adults. It was highlighted by Saltzman et al. (2020) that, unique to COVID-19, the wide access to digital technology which can buffer loneliness and isolation may also lead to greater mental health problems (Smith et al., 2018). Additionally, Sippel et al. (2015) showed the importance of social networks in promoting resilience to stress and trauma. Indeed, a wide-ranging report by the US Academy of Sciences led to Lancet (2020) calling for recognition of loneliness as a public health problem. Such highly regarded evidence does by its very existence, in turn, challenge past findings on sex work that have conflated the isolated ways in which sex workers were forced to operate, as a prima facie case that sex work was inherently harmful.

Such findings support and add to the growing awareness of that the ways in which repressive governance has forced sex workers to work in isolation were itself inherently harmful rather that the work itself. This evidence, and the subsequent discourses around sex work, needs to be reassessed in this new context and knowledge. Such reassessment, placed alongside the evidence from public health science that has been established over several decades, shows that criminalization is harmful to sex workers by forging and reinforcing health inequalities, creating contexts for violence and discrimination. Sex workers are disproportionately at risk of violence, sexual, physical and emotional harms, all linked to criminalized frameworks of governance (Rekart, 2015; Shannon et al., 2015; Goldenberg et al., 2017; Deering et al., 2014). Platt et al. (2018) conducted the first systematic review and meta-analysis of qualitative and quantitative studies published from 2000 to 2018 and found that the context of criminalization, including the threat of police arrests, raids and harassment, had significant detrimental effects on individuals (Benoit et al., 2016; Krüsi et al., 2016). Platt et al. (2018) document the ill effects of criminalization as evidenced in the 134 studies they assessed noting how sex workers are more likely to work in isolation, be disassociated from their peers and related support and experience barriers to accessing services, with limits of harm reduction. This systematic review overwhelmingly states that the evidence shows ‘extensive harms associated with criminalisation of sex work, including laws and enforcement targeting the sale and purchase of sex and activities relating to sex work organisation’. It is this standard of evidence that needs to be used so that models of work and workspaces can be the healthiest they can be and can be facilitated in a decriminalized framework.

The coronavirus pandemic casts a spotlight on scientific expertise as crucial to effective governance and policymaking, with some scientists becoming well-known personalities and trusted public figures. Importantly, it exemplified that the respect, and even awe in some cases, shown to wet-lab scientists as they isolated genomes, developed vaccines, ran drug trials and discovered mutations across the world was not shown to behavioural scientists. By contrast, social and psychological researchers were questioned, their statistical modelling and hypotheses challenged and their personal lives ‘exposed’ in newspaper stings and criticized by parliamentarians choosing to promote false binaries between economic and social behaviour (as happened to Prof Neil Ferguson of Imperial College). This attitude, by policymakers and the media, is not unknown to those researching sex work when the rigorous findings of behavioral or social scientists are placed, with faux balance, alongside those of columnists, commentators or others with vested interests. Without the noise, however, that the false equivalence between expertise and commentary brings, the necessary discussion about rights, and the best way to protect them, can move forward with a proper analysis about how people can be made safer.

It is important to note that the news media’s visions of policymakers flanked by scientists and public health experts often followed in the media with powerful life stories and narratives to help the public understand the real-world impact may help critical reflection on the role of ‘expertise’ and its relationship to ‘lived experience’ as it has become an area of mild controversy in sex work studies where confusion can arise of the difference between representativeness to enlighten, and academic research to inform, policy. This is certainly an area where the pandemic parallel can be instrumental in critical reflection on sex work research. The discourses used in such discussions, however, stem from issues that relate back to the Hart-Devlin question.

## The Hart-Devlin Question

Policy and academic discussions on sex work are often analogous to the Hart-Devlin debate which, in simple terms, equates to ‘fairness’ vs. ‘ideological morality’. Hart’s influential text of legal philosophy *The Concept of Law* (1961) made a distinction between the law and morality, and Hart’s *Law, Liberty and Morality* (1968) argued that law exists to protect individual liberty and should not be based on the popular moral consensus in the absence of harms. Devlin’s argument, on the other hand, focused on the role of the criminal law for the enforcement of morals. Devlin’s philosophy of law argued that when a behaviour reached the limits of ‘intolerance, indignation and disgust’, legislation against it was necessary. Discourse around sex work may focus on certain nation or federal states, as laws are changed, but these debates can often be reduced to the philosophical differences between pragmatists and idealists. The debate was prompted by Report of the Wolfenden Committee on Homosexual Offences and Prostitution (1957). The heart of the report was that it was not the task of the state to legislate about morality. Rather, the role of the law, was

to protect the citizens from what is offensive or injurious and to provide sufficient safeguards against exploitation and aggravation of others, particularly those who are especially vulnerable because they are young, weak in body or mind, inexperienced, or in a state of special physical, official or economic dependence. It is not, in our view, the function of the law to intervene in the private lives of citizens...

The Wolfenden Report, therefore, divided behaviour into public/private realms; we legislate the former, leaving the latter to individual liberty and decision-making. While happy with the report’s recommendations, Devlin disagreed with the philosophy underpinning it and argued that it was acceptable, even right, for the state to legislate on individual morality (Devlin, 1968). He went further to argue that if a culture agrees on a shared morality, and there is agreement on what (most) people hold a deep sense of revulsion for, then it is appropriate to make that illegal. Hart’s response was that we must distinguish between that which ‘harms others’ (public) and that which is ‘private’ and critiqued Devlin’s assumption that all morality — sexual morality, together with the morality that forbids injurious acts such as killing, or stealing — form a seamless web, so that those who deviate from one will necessarily deviate from the whole. It is Hart’s philosophy of the separability of law and morality, alongside a pragmatic approach to policy, which underpins this special section as it takes forward a range of feminist, queer and social scientific concepts and evidence with a focus on harm reduction and decriminalization. Given that the Wolfenden report was successful in partially decriminalizing homosexuality a decade later (1967) in England and Wales, its influence on sex work was less pronounced (see Morris, 2018). First, therefore, we explore how this has played out in the intersectional politics of decriminalization as it pertains to intersecting identities of gender, race and sexuality.

## The Intersectional Politics of Decriminalization

In the early twenty-first century — with notable exceptions such as New Zealand (see Armstrong; Aroney; Bond, in this special section) — legislative approaches trended toward the increased regulation of sex work. Policy advocacy in this area has focused on nebulously defined areas of concern such as online environments and global trafficking. For example, in the U.S. context, the Fight Online Sex Trafficking Act (FOSTA) and Stop Enabling Sex Traffickers Act (SESTA) criminalized websites advertising any form of sex work (see Blunt & Wolf, 2020; Mia, 2020). It followed a string of investigations by the Federal Bureau of Investigations which shut down websites including backpage.com and rentboy.com. It is possible that the reason both online sex work and sex trafficking became a focus for carceral intervention is because these could be defined as anarchic spaces — there is no ‘world government’. The internet is a shared network — which circumvents hierarchical organization. Notwithstanding the significance of digital monopolies in tech giants (e.g. Amazon, Apple, Facebook, Google and Microsoft) and global entities such as the UM, these ‘sites’ of sexuality have no identifiable individual in control, which may be a reason that they have been constructed as ‘dangerous’ or ‘shadowy’, and thus deserving of sweeping criminal interventionism. In contrast to such regulatory intervention, which can range from criminalization to legalization, calls for decriminalization recognize both the moral and practical potentials of this ‘anarchism’.

In response to the social problems of harm and exclusion, knowledge production about sexuality through research and policy discourses is always an intrinsically political process (Foucault, 1978). Political philosophies of conservatism, liberalism, feminism and so on make different claims about the role of the state in regulating and safeguarding such behaviours. Similarly, anarchism (Goldman, 1969) — sometimes referred to as libertarian socialism (Chomsky, 2013) — has also informed calls for the decriminalization of other behaviours and identities, including lesbian, gay, bisexual, trans and queer (LGBTQ) people; HIV-positive people (see Ashford et al., 2020) and people of colour, who have tended to experience disproportionate incarceration and police violence. The simple slogan ‘defund the police’ used by some Black Lives Matter campaigners, and often misunderstood in this regard, calls for the state to (re)invest resources differently, away from a carceral and militarized state, towards public goods of healthcare and education. However, it is crucial to recognize the interconnectedness of so-called radical policies to decriminalize and deregulate, shifting resources away from the apparatus of the carceral state: courts, prisons, police and military technologies (Davis, 2012; Benoit et al., 2019). As such, an anarchist interpretation of the state (or, more appropriately, against the unjust, moralizing, centralized state) is key to debates around decriminalization.

Black feminist scholarship has also been central to understanding the ways in which privilege and power intersect (Crenshaw, 2017). Alongside healthcare and justice systems, an intersectional lens has also been applied to diverse forms of sex work (Jones, 2015; Logan, 2017; Morris, 2018). For example, focusing on webcamming, Jones (2020, p. 97) has noted how online technologies have expanded work opportunities for groups often excluded from traditional labour markets, including disabled people, LGBTQ people and people of colour, where ‘camming provides a relatively stable source of income in a safe environment for people who face rampant and legally sanctioned discrimination in the economy, as well as in other institutions.’ Therefore, in this special issue, we have drawn attention to the diversity of communities involved in sex work.

Although paternalistic narratives of ‘hidden dangers’ to women and children have existed since at least the early nineteenth century, these have been widely debunked as moral panics and conspiracy theories closely aligned with antisemitism and homophobia (Cohen, 1972; Bristow, 1982; Morris, 2018). Returning to the Hart–Devlin debate and Wolfenden report, it is also important to note that the history of liberation campaigns which have called for decriminalization, legal rights and social recognition often intersect (Chateauvert, 2014; Grant, 2014; Smith and Mac, 2018). For example, the movement for LGBTQ liberation gained global recognition following the Stonewall riots of 1969, where gender and sexual minorities, many of whom sold drugs and sex, drew ‘inspiration from civil rights and women’s rights movements’ to resist homophobic, transphobic and racist policing (Morris, 2019, p. 1). As she recalls, a decade later, Leigh (1997) would invent the term sex worker, to be a more affirmative and inclusive term for erotic labour within feminist movements. In more recent decades, however, both the LGBTQ and sex worker rights movements have seen attempts to move towards assimilationism or respectability politics (Chapkis, 2017).

As discourse analysts point out, in much of daily life, we speak of abstract concepts such as ‘public health’ and ‘sexual activity’ as if everyone understands terminology in the same way and that these concepts have universal meaning (Willig, 1999), so it is with sex work. Different meanings and concepts of what constitutes sex work depend on knowledge, experiences and standpoints. For example, if someone has chosen to do that work and self-defines as a sex worker, then it is their right to define, redefine or reject the terms on which such a label is understood. Beyond that, there are many areas where terminology is more contested. For example, is it ‘stripping’ or ‘lap dancing?’ Is it work carried out for a specific length of time? Recent work on ‘incidental sex work’ (Morris, in this special section) illustrates that men on platforms such as Grindr, who may have exchanged sex for money or goods, question how much or what type of ‘work’ would define one as a sex ‘worker’, as do others such as findommes (see Brooks-Gordon and McCracken, in this special section) who benefit financially from clients but do not identify as ‘sex workers, or people receiving illicit substances for sex at Chemsex parties’ (Brooks-Gordon & Ebbitt, 2021; 5). In the criminal law, a subjective test is used, i.e. something is sexual if the person involved defines it as such, as seen in common law cases of foot fetishism (Bainham & Brooks-Gordon 2004). In statues (inter alia The Sexual Offences Act, 2003), there are definitions about the type of payment that constitutes benefit. A sex worker is therefore someone who benefits financially from labour that their clients perceive as sexual — it is this definition with which we tentatively proceeded and also seek to explore. Sexual minorities have arguably always had to contend with the tensions between ‘fitting in’ with social norms, something which has been characterized as a ‘successful’ strategy for the LGBTQ rights movement, contrasted with forms of direct action which could be characterized as ‘queer’ in the postmodern sense of this term (Adler, 2018).

This tension also occurs at the level of ‘identity politics’, where policy is often shaped by which checkboxes a person chooses, or is able, to tick in the clinic or the courthouse. In deregulated spaces such as the internet, much has been written about the expanding variety of expression and identification which move beyond the gender binary or traditional categorizations, definitions and labels of sexual identity (Weeks, 2017). For example, how does the state classify and care for those who sell sex incidentally (on platforms such as Grindr or OnlyFans) but would not identify themselves as sex workers (see Morris, in this special section). This trend further complicates an essentialist focus on ‘women and girls’, usually constructed as vulnerable and victimized, and therefore without agency. This too can construct issues around who gets to speak ‘about’ and ‘for’ sex workers, especially when some of those defined as such by law, policy and research may not recognize that designation. It is an issue with a long history within the global sex worker rights movement, dotted throughout history, but emerged in the 1970s from the sex-positive part of the radical feminist movements that gave voice to women’s sexual choices and right to determination over their bodies has a large part to play in our understanding of decriminalization.

In the USA, the setting up of COYOTE (Call Off Your Old Tired Ethics) by the late Margo St. James in 1973 was a call to arms across the world to galvanize, organize and politicize the issues faced by sex workers. The World Charter for Prostitutes Rights (1985) further set out the core parameters of laws, human rights demands, working conditions, health, services, taxes and public status of sex work as a benchmark for change (see Pheterson, 1989). The early actions were the start of the global sex worker rights movement and coupled with labour right movements that often harboured these issues have been present across much of the globe. Crago (2008) presented the activities of organizations in Bangladesh, Brazil, Canada, India, Russia, Slovakia, South Africa and the USA who have been working tirelessly for decades to campaign for (and often provide) public health initiatives and rights for sex workers. Protests against the globe to defend the human rights of sex workers against incarceration, violence, eviction, exploitation, humiliation and extortion have been the work of global organizations such as SWAN (Sex Worker Rights Advocacy Network for Central, Eastern Europe and Centra Asia), ICRSE (International committee for the Rights of Sex Workers in Europe) (ICRSE, 2021) and APNSW (Asia Pacific Network of Sex Workers). Individual nations have an abundance of service delivery NGOs and activist groups that have decriminalization at the core of their mission (see Jackson, 2019 and ECP, in this issue). We can learn greatly from these collectives and community strategies around the world, such as those explained in India by Dasgupta and Sinha in this issue. Therefore, drawing on Foucault (1978), this special issue recognizes that ‘policing’ is something which also happens beyond the disciplinary apparatus and machinery of the carceral state: the media and educational and healthcare systems have significant power to shape discourse and ideology in exclusionary ways. The regulatory role of the state also goes beyond punishment, including reforms to welfare and taxation (see Bateman, this issue).

## Global Health and Human Rights Campaigns

As we noted above, the COVID-19 pandemic — not unlike the HIV/AIDS pandemic — has brought the importance of global public health to the attention of the media, public and international organizations which have long advocated for the decriminalization of sex work on health grounds. For example, the Director-General of the World Health Organization (WHO), Tedros Adhanom, has relayed important biological and medical information to global audiences. This gives renewed weight to work done previously by the WHO, particularly to assist with HIV prevention and treatment, and as a means to promote human rights, including for sex workers:

WHO supports countries to address these structural barriers and ensure sex workers’ human rights as well as implementing a comprehensive package of HIV and health services for sex workers through community lead approaches. (WHO, 2021)

In 2012, the WHO made a clear statement which recommended that ‘countries work towards decriminalization of sex work and urge countries to improve sex workers’ access health services.’(WHO, 2015). This was followed in 2016 by Amnesty International, which after comprehensive global consultation adopted a decriminalization policy for sex work, stating that

It recommends the decriminalization of consensual sex work, including those laws that prohibit associated activities—such as bans on buying, solicitation and general organization of sex work. This is based on evidence that these laws often make sex workers less safe and provide impunity for abusers with sex workers often too scared of being penalized to report crime to the police. Laws on sex work should focus on protecting people from exploitation and abuse, rather than trying to ban all sex work and penalize sex workers. (Amnesty, 2016)

The Amnesty International policy, with its measured and forensic analysis of human rights, was a major boost to sex worker activists and campaign groups across the world, as it endorsed their lived experiences (and decades of campaigning) as a reliable source of evidence to promote legislative and policy change, supporting the work of the WHO. Other organizations which openly support the decriminalization of sex work include the Global Commission on HIV and the Law, the UN Special Rapporteur on the Right to Health, Canadian HIV/AIDS Legal Network, Human Rights Watch, the Kenya National Human Rights Commission, the Bill and Melinda Gates Foundation, the International Commission of Jurists, the Joint United Nations Programme on HIV and AIDS, the Office of the United Nations High Commissioner for Human Rights, the Open Society Foundation, Human Rights Watch (Human Rights Watch, 2019) and the South African Commission on Gender Equality. The knowledge, particularly around the implications for safety and human rights, has seen traction in some key global organizations as policies and statements that support decriminalization have been adopted alongside the development of this evidence base. However, there is still a significant gap between policy proposals at the international level, legislators and decision-makers and public opinion. We now turn briefly to consider further where public opinion sits.

## Public Opinion Trends Towards Decriminalization

A trend towards social liberalism in public opinion has continued for over 30 years on sexual relationships and sexual behaviour according to the British Social Attitudes Survey (BSA, 2019a, b). The long-term increase in socially liberal attitudes since the BSA began in 1983 and has accelerated recently with this year’s BSA list of questions including the use of pornography and transgender rights. Public opinion on personal freedom and the continued rise of social liberalism extend to a public in favour of decriminalization when polled along scientific principles — even though most polls are carried out when bills are introduced. For example, YouGov polled voters in the USA where New York lawmakers introduced bills on decriminalization and in one of the largest polls on the issue ever, with 175,41 adults polled on the issue of allowing paid sex between consenting adults while maintaining prohibitions on trafficking, coercion and sexual abuse of minors (YouGov, 2019). In 2020, the thinktank Data for Progress polled voters aged 18 to 44 years of age, of which two thirds supported decriminalization and across all age groups an outright majority support decriminalization. In the US primaries for President, many of the candidates ran for election on a ticket of decriminalization, as the growing consensus among civil rights; LGBTQ+ and justice and worker/labour rights; immigration justice and women’s groups in the USA recognize that it is the best way to keep people safe as part of an effective anti-trafficking strategy and the best way to service the needs of people in sex work and promote racial, gender and economic justice (Data for Progress, 2020). As many campaign groups are beginning to find out, there is solid support for decriminalization. For example, the campaign group Decrim finds that support rises to a 3 to 1 margin in voters of left-leaning views such as democrats in the USA (Decrim, 2019). In the UK, more Britons support decriminalization than oppose it, for example in a poll of 2000 people by Survation, 49% support decriminalization of the brothel keeping laws which prevent sex workers safely working together (Each Other, 2019). These public insights build on the corpus of research and commentary that reflects the mainstreaming of sexual consumption and labour in the west (Brents & Sanders, 2010).

With all the evidence in mind, in this special issue, we ask how rights can be protected within admissive statutes. What would a good sex work law look like? What would be a general framework for policy on commercial sex? The exploration moves to explore *how* the decriminalization of sex work might work in practice. The aim is to consider new models of practice, problem solving and harm-reduction in a contemporary context. This special issue explores evidence-informed policy proposals which move beyond false debates by focusing on the diversity of sex work, public health and harm reduction and which includes an examination of what policy would look like in a decriminalized context, something which is often speculative, as few countries have adopted this (see Armstrong; Bond, this issue).

Keeping these issues in mind, a special acknowledgement here must go to the work of the New Zealand Prostitutes Collective because of their unwavering determination to work with allies such as politicians in 2000+ which saw the passing of law reform to a decriminalized model (see Aroney this issue) and also to the Durbar Mahila Samanwaya Committee in Kolkata, India, who have advocated for the rights of the 65,000 sex workers of Sonogachi working against trafficking and HIV since 1992 (see in this issue Dasgupta and Sinha). So, it is well established that decriminalization is the supported model for better health outcomes, reduced violence and is backed by a range of global entities and has been at the centre of grassroots campaigns from the sex work community for some time.

## Universal Principles

At the time of writing, we mourned the loss of Margo St James — a sex-positive feminist who pioneered the sex worker rights movement in the 1960s in San Francisco, establishing COYOTE at the start of the modern decriminalization movement across the globe. In her memory, and of those who have gone before and after her, we return to some key principles of rights to reiterate, reinforce and remember as we introduce the articles for this special issue. We hope the special issue will draw out the ways in which structural stigma is experienced and can be overcome (see Bruckert & Hannem, 2013; Krüsi et al., 2016; Benoit et al., 2018) and speak to the debates about how to improve and move forward sex work policies (see Overs & Loff, 2013; Abel, 2019).

If the underlying point of decriminalization is harm reduction and safety, and the overlying point is the exercise of rights at work, then we need to explore the environment in which this work takes place — and all the papers in this special section do so to a greater or lesser extent. In creating a safe world, there are minimum standards on food, water, air quality and so on. While there are disagreements amongst politicians about how achievable various standards is, there is a level of consensus amongst scientists about what constitutes minimum standards. What would one want to see in a decriminalized working environment where the documented injustices would not prevail (see NSWO, 2020)? Barriers for entry cannot be set too high; otherwise, they prevent new entrants (especially those who previously worked for larger enterprises to work for themselves). Such barriers can be seen in Nevada, in the Netherlands with the introduction of regulations for licenses that migrants were not allowed to apply for and with the sexual services establishment licenses in the UK (Hubbard & Colosi, 2013; Sanders & Campbell, 2013; Pitcher & Wijers, 2014).

In thinking what needs to be done to obtain rights for sex workers, and improved living and working conditions, there is history on which to build. The consensus around various statements and charters across the globe covers these key rights: the right to associate and organize, the right to be protected by the law, the right to be free from violence, the right to be free from discrimination, the right to freedom from arbitrary interference, the right the health, the right to move and migrate, the right to work and free choice of employment. The global international movement, the Network of Sex Work Projects, promotes these rights which have a set of core foundations: acceptance of sex work as work, opposition to criminalization and support for self-organization and self-determination. As Ostergen (2017) notes, law reform to decriminalization is one state of inclusive citizenship, and other key elements such as the living wage, improved police relations and inclusive housing are essential. We hope the articles in this special issue continue to build on this existing knowledge regarding what would be the general framework of conditions, rights and principles. The papers we have selected for this special issue focus on both the lived experience of sex work and also the premises on which governance is built.

## Summary of Special Section Papers

Comparing sex work with care work, Bateman’s paper calls into question the double standards of ‘radical feminists’ who seek to abolish the former for perpetuating gender inequality but do not demand the abolition of care. She provides a historical overview of how the ‘immodest woman’ has been constructed, contributing to contemporary ‘end demand’ approaches, and how it is ‘whorephobia’—rather than sex work—which harms all women.

McCracken and Brooks-Gordon’s paper explores the nature of online work by financial dominitrices or ‘findommes’ working via video call and webcams. In a large two-stage survey, they investigate the experience of 195 findommes on money-slavery websites and social media. Using netnography as a tool, their analysis reveals how findomme interaction progresses from text-based interaction to virtual face-to-face and voice communication to show financial domination on a continuum from being a lifestyle choice in the BDSM community that reaps financial benefits to a purely economic and legitimate form of commercial labour.

Their findings also show how findommes dictate and create their boundaries and maintain psychological health and enhance our understanding of how the microculture of findomming interacts with other micro-cultures in a paper that adds to the growing body of literature that destigmatizes consensual erotic labour.

Morris combines queer theory with empirical data in the first study of incidental sex work, drawing on mixed methods including interviews with 50 young men who agreed to sell sex on social media platforms without advertising or identifying as sex workers and an incidental survey of 1473 Grindr users aged 18 to 28, in cities across England and Wales, raising important questions about the limitations of identity politics for making sense of the diversity of sex work practices and campaigns for sexual minority rights.

Whitney Berry and Frazer’s paper provides a compelling comparison that sex workers make when a statute changes their everyday lives and lived experience. From interviews with sex workers in the Republic of Ireland following the introduction of punitive legislation, themes emerged that vividly highlight the differences in mental health, police interaction, housing, family relationships, client interaction, discrimination and relationships with other sex workers. Such in-depth analysis provides a rigorous assessment that illustrates that sex work legislation in Eire has failed in a stated mission to improve life for sex workers, and that other options, such as decriminalization, should now be considered.

Bowen and Swindells’s paper combines a survey of 88 sex working members of the National Ugly Mugs in England and with their data on victimization to provide insights on the factors that deter sex workers from involving police as part of their justice-seeking efforts.

The survey results demonstrate a disturbing trend of sex workers feeling alienated and distrusting of police and the courts. The implications of sex workers not sharing information about dangerous individuals with police and choosing not to participate in court processes signal significant flaws in our criminal justice system regarding safe and inequitable access and poses danger to everyone as violent men go free.

Benoit centres the voices of sex workers in her article, using the responses of 57 participants to ask what changes are needed to improve health, safety and rights under a criminalized system in Canada. Findings firmly advocate for decriminalization and for health and welfare policies to shoulder the governance of sex work.

In the European context, Henham’s comparative fieldwork in 2016 draws on interviews and observations of visible sex work spaces across ten cities in Belgium, France, Germany, the Netherlands and Switzerland, demonstrating the limitations of simplistic punitive legal or policy approaches noting both unexpected benefits and consequences of different regulatory regimes, alongside how changes which have occurred in the context of COVID-19 may offer an indication of what further criminalization might look like.

In an Asian context, Dasgupta and Sinha combine ethnographic studies between 2009 and 2016 to share findings of the impact of criminalization on sex workers in Kolkata, India. Their article shines a light on the resistance movements led by sex workers who try to mitigate harms through community-based strategies, NGO stakeholders and partnerships and self-regulatory bodies. Estacio, Alibudbud and Zsanila Estacio reflect on drugs users in the Philippines who are engaged in sex work to explore the stigma of HIV, drug use and selling sex. Their results construct an argument that calls for a shift to public approaches and community-based programs to address the needs of this group.

We are very pleased to have three papers which speak from New Zealand — the only country that has decriminalized sex work — where global scrutiny turns to see law reform, implementation and operation in action. Armstrong’s paper draws on the concept of zemiology (social harm), alongside interviews with 46 sex workers in New Zealand, noting how this country provides an exceptional case study to explore how decriminalization has worked in practice to improve the autonomy and quality of life experienced by sex workers. Aroney’s longitudinal research from 2012 to 2019 with a range of stakeholders traces the important journey that saw the development and implementation of the Prostitution Reform Act (2003) in New Zealand. The focus here is on how and why sex workers and their allies can work together for law reform, with sex workers taking the lead in educating. Bond’s paper coins the ‘Dunedin model’ revealing a specific place in New Zealand and examines what decriminalization can look like under localized interpretations around brothel organization. This paper speaks to the necessity of processes and key stakeholders needed to ensure that decriminalized laws are fully operationalized.

In combination, this special section provides an examination of sex work policy and research across 12 countries in four continents, providing an important space for international comparisons. The research represented here supports calls by global health and human rights organizations for sex work decriminalization as the best strategy to reduce harm.
